# Registry-based randomised clinical trials: a remedy for evidence-based diabetes care?

**DOI:** 10.1007/s00125-022-05762-x

**Published:** 2022-07-29

**Authors:** Jan W. Eriksson, Björn Eliasson, Louise Bennet, Johan Sundström

**Affiliations:** 1 Department of Medical Sciences, Clinical Diabetes and Metabolism, Uppsala University, Uppsala, Sweden; 2 Department of Medicine, Sahlgrenska University Hospital, Gothenburg, Sweden; 3 Swedish National Diabetes Register, Västra Götalandsregionen, Gothenburg, Sweden; 4 Department of Clinical Sciences in Malmö, Lund University, Lund, Sweden; 5 Clinical Trials Unit, Skåne University Hospital in Lund, Lund, Sweden; 6 Department of Medical Sciences, Clinical Epidemiology, Uppsala University, Uppsala, Sweden; 7 The George Institute for Global Health, University of New South Wales, Sydney, NSW Australia

**Keywords:** Clinical outcomes, First-line treatment, Glucose-lowering drugs, Healthcare registry, Macrovascular complications, Microvascular complications, Mortality, Randomised trial, Review, Type 2 diabetes

## Abstract

This narrative review describes a new approach to navigation in a challenging landscape of clinical drug development in diabetes. Successful outcome studies in recent years have led to new indications and guidelines in type 2 diabetes, yet the number of clinical trials in diabetes is now declining. This is due to many environmental factors acting in concert, including the prioritisation of funding for other diseases, high costs of large randomised clinical trials, increase in regulatory requirements and limited entry of novel candidate drugs. There is a need for novel and cost-effective paradigms of clinical development to meet these and other challenges. The concept of registry-based randomised clinical trials (RRCTs) is an attractive option. In this review we focus on type 2 diabetes and the prevention of cardiovascular and microvascular comorbidities and mortality, using the Swedish SMARTEST trial as an example of an RRCT. We also give some examples from other disease areas. The RRCT concept is a novel, cost-effective and scientifically sound approach for conducting large-scale diabetes trials in a real-world setting.

**Graphical abstract:**

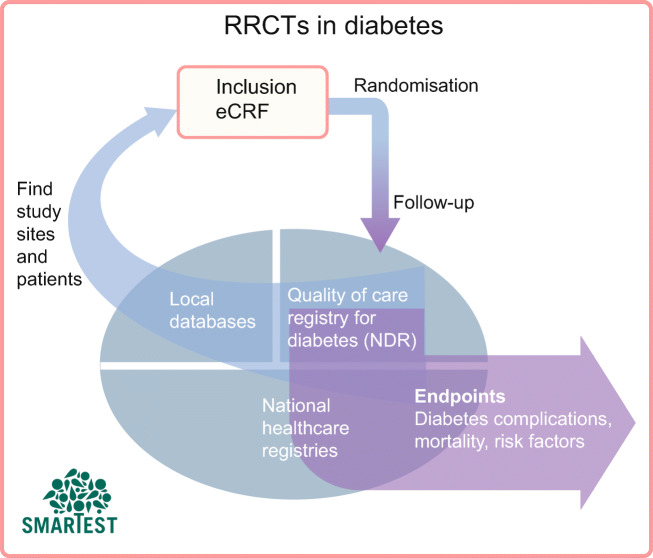

**Supplementary Information:**

The online version contains a slideset of the figures for download, available at 10.1007/s00125-022-05762-x.

## Introduction

**Fig. 1:**
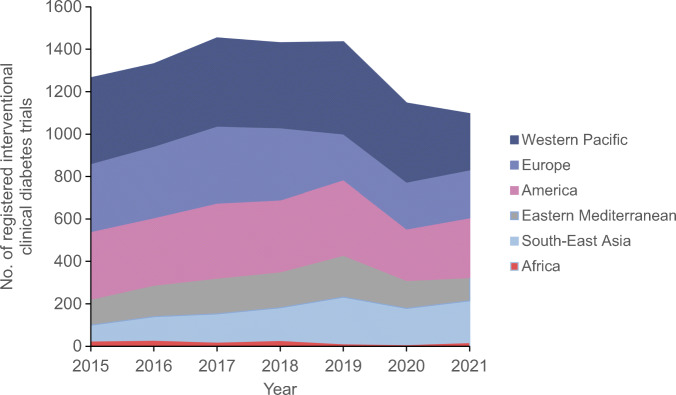
Number of registered clinical trials in diabetes worldwide, 2015–2021. Data were obtained from the WHO [1]. This figure is available as part of a downloadable slideset

The number of RCTs in diabetes has increased over the last two decades. According to WHO data, most studies between 1999 and 2021 were conducted in the USA (24%), Japan (11%), Germany (9%), India (8.5%), the UK (8%) and China (7%) [1]. There has been unprecedented success in demonstrating long-term improvements in cardiovascular and renal clinical outcomes as well as survival rates in type 2 diabetes with the modern glucose-lowering agents glucagon-like peptide-1 (GLP-1) receptor agonists and sodium–glucose cotransporter 2 (SGLT2) inhibitors. This has been achieved by performing numerous large outcome trials, mainly in type 2 diabetes patients with established cardiovascular or renal disease. However, since 2018 the number of trials has decreased globally, except in South-East Asia (Fig. 1). In Sweden, the number of trials initiated was halved between 2004 and 2016 [2]. The reasons for this declining trend may be related not only to existing healthcare infrastructure and resources and increases in regulatory requirements for safety and efficacy documentation, but also to new legislation and funding environments, such as following Brexit in the UK [3]. Diabetes trials may also be affected by pharmaceutical companies prioritising other disease areas. In the long-term perspective, a decline in clinical trials may preclude patients with diabetes from taking advantage of new effective pharmaceuticals.

In most European countries, people with type 2 diabetes are primarily diagnosed, treated and followed up in primary care [4, 5]. Thus, to ensure selection of representative study samples, clinical trials addressing people with type 2 diabetes should ideally take place in primary care settings. However, there are challenges connected to deficiencies in budget, staffing and experience. Incentives to encourage the allocation of time to recruit participants and conduct clinical trials are limited. Most primary care centres lack good clinical practice (GCP)-trained staff, and the turnover of primary care physicians is often high. This may obstruct the participation of people with type 2 diabetes in long-term follow-up studies.

There are still significant gaps in our toolbox for supporting the selection of optimal treatment(s) for each individual with type 2 diabetes, as highlighted by the *Lancet* Commission on diabetes [6]. Hence, a new paradigm for cost-effective clinical trials in diabetes is warranted. The registry-based randomised clinical trial (RRCT) concept is a novel, pragmatic clinical research option that can substantially cut costs and allow large trials to be conducted in regular healthcare settings. Here, we present the nuts and bolts of the RRCT concept and focus on the potential of RRCTs to evaluate new therapeutic strategies, in particular in type 2 diabetes. Some pioneering examples from various disease areas, including type 2 diabetes, are provided, obtained by searching PubMed for ‘registry-based trial’ and ‘registry-based trial and diabetes’ and screening for diabetes trials at https://clinicaltrials.gov (accessed 31 Jan 2022). This work is not a systematic or complete literature review. Instead, it focuses on recent examples of the use of healthcare registries and the novel RRCT study design. A main purpose is to encourage and stimulate future large-scale clinical trials in the diabetes arena, including in times of increasing societal, financial and regulatory challenges.

## Registry-based randomised clinical trials: rationale and scope

Optimally, all questions addressing the relative effects of interventions would be studied using a randomised approach [7, 8]. In lieu of this possibility, methods for observational comparative effectiveness research are rapidly advancing as healthcare databases become increasingly detailed, structured and available. One such data source is quality-of-care registries [9], which typically are infrastructures initiated by healthcare professionals with the purpose of improving adherence to clinical care guidelines. Such registries are usually based on reports of individual data on disease history, treatments and outcomes of all comers in a particular clinical field. In many cases, open comparisons of the delivery and results of clinical care between healthcare units are also provided.

The strengths of observational comparative effectiveness studies within quality-of-care registries include their low costs, generalisability to everyday clinical care, ability to capture interventions as they are actually delivered, broad participant populations and large sample sizes, providing opportunities to identify rare and late events. Weaknesses include the variable and uncertain data quality and risk of bias, There are several examples of observational registry-based studies in Scandinavian and other Western European countries addressing the comparative effectiveness of type 2 diabetes treatments [10, 11], but very few in other regions.

In contrast, RCTs provide unconfounded effect estimates, but their weaknesses include the high or even excessive costs for trial operations, narrowly selected samples and treatment protocols with questionable generalisability to clinical reality. Several large-scale cardiovascular outcome trials in type 2 diabetes have shown low representativeness of participants regarding demographic composition, comorbidities and diabetic complications compared with patients in primary care [12]. This diminishes the generalisability and relevance of results from these trials in relation to real-world settings [13–15]. Interestingly, differences in the composition of study populations may become evident when analysing a comparative effectiveness observational study and a traditional RCT in parallel. Such comparisons suggest that traditional RCTs often have less representative study cohorts, and this can also involve the distribution of endpoint measures [10].

Pragmatic trial initiatives to overcome the drawbacks of both observational studies and ordinary RCTs have been proposed for more than half a century [16] but have been implemented only in the last decade following the emergence of quality-of-care registries and the possibility of registering pragmatic trials within them. RRCTs have the advantages of both the generalisability of the study samples and the confounding control of the RCT. While the aim of a traditional RCT is typically to study the efficacy of a treatment in selected participants under ideal circumstances, RRCTs aim to study the effectiveness of treatments in real-world patients under normal everyday circumstances [16]. The quality-of-care registry may support some or most parts of a clinical trial, depending on the maturity of the registry and the nature of the research questions. It can be used to identify suitable study sites and eligible participants, provide and collect consent forms, perform randomisation, collect baseline characteristics and, most importantly, capture clinical endpoints.

### The development of pragmatic registry-based randomised clinical trials

RRCTs have been performed in gynaecology, orthopaedic surgery, bariatric surgery and pulmonary medicine [17–22]. The pioneers in RRCTs, however, were researchers using the Swedish Web-system for Enhancement and Development of Evidence-based care in Heart disease Evaluated According to Recommended Therapies (SWEDEHEART) [23]. One reason for the initial usefulness of this quality-of-care registry for clinical trials is that the percutaneous coronary intervention part of the registry is used online, that is, staff interact with the registry while the patient and physician are in the same room. Thus, individual patient data entered into the registry are continuously and automatically checked against inclusion criteria for trials. Suitable trials can be proposed in real-time and patients can be easily included. The first RRCT using SWEDEHEART was the Thrombus Aspiration in ST-Elevation Myocardial Infarction in Scandinavia (TASTE) trial [24], which randomised participants to either undergo coronary thrombus aspiration or not. It was extremely cost-effective as only a randomisation module was added to the usual components of the registry. The one-shot nature of the treatment did not demand longer-term safety follow-up and the outcome, all-cause death, could be obtained from the national population registry with no loss to follow-up. Later RRCTs using SWEDEHEART have explored other trial-specific additions needed for out-of-registry inclusion procedures [25] or additional safety follow-up [26]. The ease and speed of inclusion of participants into such trials is evident, as two out of three eligible participants nationwide were rapidly randomised into two of the trials [24, 26]. In contrast, an American trial with a similar research question had to be terminated prematurely because of slow enrolment [27].

Although healthcare registries have been used extensively for observational treatment studies, anchoring an RRCT to one or more registries is a novel approach. However, although pragmatic trial components have been reported historically [28] and recently [29], to date there has been very limited international implementation of the RRCT approach outside the Nordic countries [30, 31]. A prerequisite for RRCTS is timely access to high-quality registry data that can be used to determine if patients meet inclusion/exclusion criteria and/or to obtain efficacy and safety outcomes.

Notably, medical product authorities currently do not view RRCTs differently from ordinary RCTs and the regulatory demands are identical [32]. However, certain literal interpretations of GCP will hamper implementation of RRCTs [33]. An approach to GCP that values trial efficiency and completion, while maintaining adequate risk management and regulatory compliance, is crucial in both sponsor and contract research organisations. It is paramount to use data sources with high coverage that include effect and safety variables that can be reliably validated. Outcomes collected from registers should ideally include safety data with time intervals that are short enough for safe trial conduct. Otherwise, alternative procedures will be needed, for example targeted collection of serious adverse events. When outcome data are to be collected from registries, it is particularly attractive to launch a trial within a healthcare system that covers a representative target population and provides standardised follow-up data. In this regard, data from national healthcare registries that are available for research purposes are very useful. Nonetheless, health maintenance organisation and similar claims databases can also be used. For all healthcare databases, their utility will depend on their data quality and structure, their proximity to patients, healthcare providers and researchers and their coverage over geographical regions and time.

### When is a registry-based randomised clinical trial the right tool?

Table 1 summarises the advantages and disadvantages of RRCTs and traditional RCTs. In brief, a traditional RCT is probably preferable when the study involves a small participant group, non-approved investigational treatments, intense safety monitoring and/or the need for frequent on-site follow-up or specialised assessments of endpoint measures (e.g. biochemical, physiological or imaging assessments). RRCTs may be the design of choice for studies involving large patient cohorts, approved and well-documented treatments and simple and established endpoints that are available in registers (e.g. hospitalisations and deaths) or in normal healthcare settings (e.g. anthropometry and routine clinical biochemistry).

**Table 1:** Advantages and disadvantages of RRCTs and traditional RCTs performed in normal clinical care and in designated study centres, respectively

	RRCTs	RCTs
Characteristics favouring RCTs	Potential bias because of (usually) open label treatment	Minimal bias; masked treatment
	Only interventions with low safety surveillance needs	Interventions with unknown safety profiles can be studied
	Variable delivery of interventions as in routine care	Controlled delivery of fixed interventions
	Low control of adherence and compliance	Strict monitoring of adherence and compliance
	Varying quality of registry-based outcome data	Standardised and detailed outcome collection^a^
	Varying resources, competence and study experience at study sites	Well-educated, experienced study staff
	Varying methods for laboratory measures	Standardised laboratory measures
	Feasible mainly in selected jurisdictions	Can be performed in most regions
Characteristics favouring RRCTs	Generalisable; high external validity	Not generalisable; low external validity
	Real-world clinical setting	‘Artificial’ setting
	Participants are ‘all comers’	Participants are highly selected
	Low cost^b^	High cost
	Simple delivery of study drugs^c^	Complex delivery of study drugs, on-site; double-masking
	Performed in normal clinical care; decentralised	Requires experienced study sites and staff
	Automated data collection	Labour-intensive individual data collection
	Stimulates quality improvements in routine clinical care	No immediate impact on clinical care
	Broad healthcare involvement	Only specialised trial centres are involved
	Fast recruitment	Recruitment often challenging and slow
	Consent procedures may be simplified (e.g. electronic)	Consent procedures are standardised and tedious

^a^For outcomes that are obtained at trial centre visits but not necessarily for outcomes based on health records or laboratory analyses

^b^The cost of the SMARTEST trial [34] has been estimated to be about 10% of the cost of a traditional RCT addressing a similar research question

^c^Typically, study treatments are not masked in RRCTs; this is, however, possible but has higher costs and a higher degree of complexity

Compared with traditional RCTs, RRCTs can be faster, massively cheaper, more decentralised and time-effective. They can be performed over a large geographical area with a high number of participants. This can promote both cost-efficiency and representativeness and increase statistical power. Study treatments in an RRCT are typically, but not necessarily, unmasked to participants and their local healthcare staff (but not to researchers), and this simplifies distribution of study drugs. As prospective studies require individual identifiers for all study participants, RRCTs can most easily be carried out in selected jurisdictions where individual social security numbers provide basic identifying information and are a natural part of society’s administrative system, such as in Nordic countries.

**Fig. 2:**
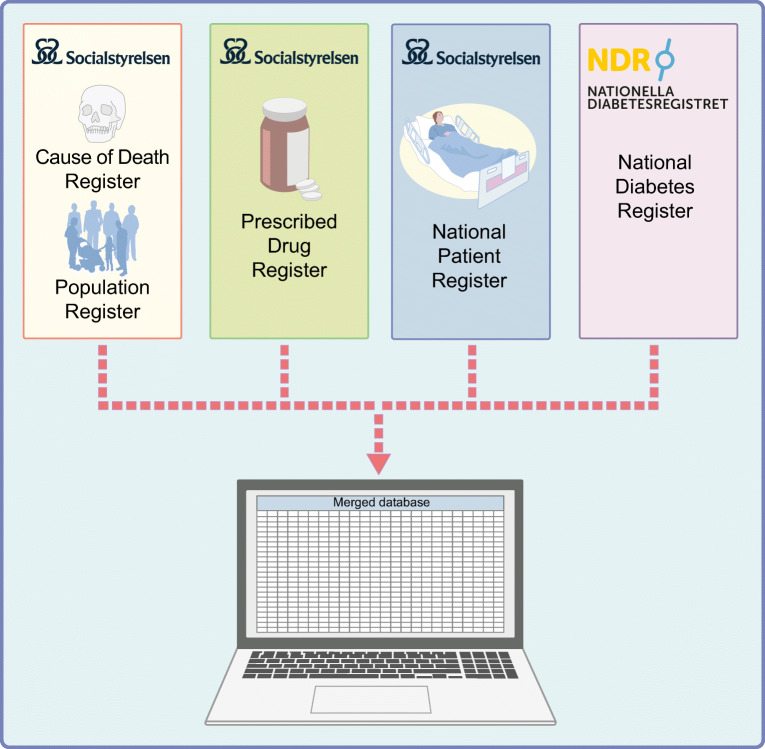
Data capture and flow in an RRCT. Examples of sources of data in an RRCT using multiple registries, here exemplified by the SMARTEST trial [34]. Socialstyrelsen, Swedish National Board of Health and Welfare. This figure is available as part of a downloadable slideset

Planning for an RRCT can be even more complex, time-consuming and demanding than planning for an ordinary RCT. Data management procedures are critical, as RRCTs need to use non-traditional trial data sources, and determining the quality and usefulness of such data is key (Fig. 2). To complement the data collection that is possible in quality-of-care registries, there is often a need to set up new tools and processes for effective trial conduct. These can be generic and facilitate multiple trials, or trial-specific; in any case, the costs of and barriers to setting them up need to be factored in early. Digital tools provide multiple benefits [33]. By allowing for remote inclusion visits by video appointment, the participation of underserved and non-traditional study participants and locations is possible, providing a broader and more representative recruitment base. Digital communication also has the potential to maximise participants’ understanding of trials and it allows for remote monitoring of informed consent, saving time and money.

As the interactions between investigators and study participants are typically sparse in RRCTs, safety surveillance in these trials is a notable focus of regulatory authorities. Ensuring that study participants provide up-to-date contact details to investigators, the development of additional safety follow-up procedures [26] and regular subscription to comprehensive adverse event data [34] may be necessary safety precautions. Ethics authorities have typically not raised higher barriers for RRCTs than for other RCTs, but we have noted a focus on ensuring that integrity issues (e.g. from cross-linking of registries) are sufficiently described in participant information. Privacy legislation in some countries or regions may raise the barriers for effective trial conduct, and exploring the utility of explicit participant consent for such data handling may be important.

**Fig. 3:**
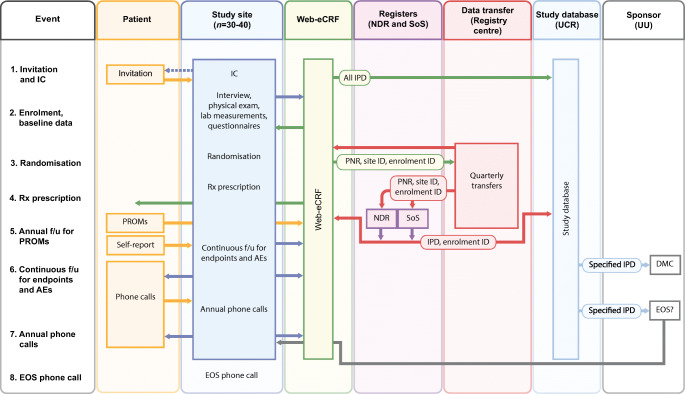
Procedures for data capture and management in an RRCT. This example is from the SMARTEST study [34] and illustrates that data flow can be complex and requires careful planning. The NDR is a quality-of-care register and is used to obtain endpoints. The Swedish Board of Health and Welfare (Socialstyrelsen [SoS]) administers national healthcare registries, used for obtaining endpoints and adverse events. AEs, adverse events; DMC, data monitoring committee; eCRF, electronic case report form; EOS, end of study; f/u, follow-up; IC, informed consent; IPD, individual patient data; PNR, personal ID number; PROMs, patient-reported outcome measures; Rx, randomised treatment; UCR, Uppsala Clinical Research Center (an academic clinical research organisation run jointly by Uppsala University and Uppsala University Hospital); UU, Uppsala University. This figure is available as part of a downloadable slideset

Even though the RRCT model can usually easily be launched in daily healthcare settings, including primary care centres, it must be acknowledged that various types of expert support are required. This may include support for central study management, monitoring of sites and participant safety, database development, data capture and analysis, and regulatory matters (Fig. 3). The expert support needed to conduct an RRCT will depend on the objectives, participant population, interventions and outcomes of the specific trial. Hitherto, RRCTs have largely been conducted with academic sponsors; however, such studies in diabetes, as well as other fields, could equally well have a company sponsor, which may reinforce appropriate compliance with GCP guidelines and provide monitoring of and other expert support for inexperienced study sites. A take-home message is that the on-site work can resemble everyday clinical practice, whereas the central study management will be more similar to that in any RCT.

## Use of healthcare registries in diabetes trials

Healthcare registries have long been used for observational studies, but carrying out an RCT within a registry is a novel methodology. We are not aware of any RRCTs that are completed, ongoing or being planned in the field of diabetes (https://clinicaltrials.gov; accessed 31 Jan 2022) except for the SGLT2 Inhibitor or Metformin as Standard Treatment of Early Stage Type 2 Diabetes (SMARTEST) trial (EudraCT 2019-001046-17, Clinicaltrials.gov NCT03982381) [34]. This trial can serve as an illustration of a possible design for an RRCT, including for recruitment, documentation and data collection of outcome variables. Crucial to this specific study are the Swedish National Diabetes Register and the nationwide healthcare and population registries, which contain information on medical conditions, drug treatments and deaths, with full national coverage (Fig. 2).

### Swedish National Diabetes Register

The St Vincent Declaration recommended several measures to improve the quality of diabetes care and to reduce the burden of complications caused by diabetes. Among these was a need to ‘establish monitoring and control systems using the latest information technology, for quality assurance of diabetes care and for laboratory and technical procedures in diabetes diagnosis, treatment and self-management’ [35]. In Sweden, the legislation also stipulates that the quality of healthcare must be developed and secured systematically and continuously. Taken together, such recommendations and guidelines prompted the initiation of the Swedish National Diabetes Register (NDR) in the first half of the 1990s and its gradual development since then.

The original purpose of the NDR was to enable the follow-up of care units’ treatment results annually, both at individual level and at unit level, providing the possibility to compare results regionally and nationally. Clinical information, risk factor levels, treatments and the presence of diabetic complications have been reported since 1996. Initially, paper forms or computer disks were used to collect data, but presently the clinical data of at least two-thirds of participants are reported directly and securely from electronic medical record systems, and the remainder are entered manually online (https://www.ndr.nu; accessed 8 February 2022). The estimated total coverage of people with diabetes was 88% in 2020.

Several scientific studies based on NDR data have been conducted, The first studies were descriptive, presenting the registry and the presence of risk factors in participants, such as hypertension, microalbuminuria, smoking and obesity [36], but subsequently several studies have addressed the links between risk factors and macrovascular complications [37, 38].

### National healthcare registries

The Nordic countries largely share history, culture and political systems as well as the organisation and financing of healthcare. Each citizen has a unique personal identity number that is used in public administration systems. This has recently been reviewed and examples of registry-based research in these countries have been provided [39]. In Sweden, there are numerous official registries and databases that are managed by authorities on behalf of the government. The quality and protection of these data are secured by laws, statutes, anonymity, confidentiality and modern information technology [9]. This includes compliance with the General Data Protection Regulation (GDPR), which has been effective in all EU member states since 25 May 2018.

There are several Swedish registries mandated by the government that have national and complete coverage and may be useful for research in healthcare and medicine. They include the Prescribed Drug Register and the National Patient Register [40, 41]. The former contains data on all prescribed drugs dispensed at pharmacies and the latter contains information on the dates of all hospital visits or inpatient stays, as well as diagnoses and procedures according to ICD-9 (http://www.icd9data.com/2007/Volume1/default.htm) or ICD-10 (http://apps.who.int/classifications/icd10/browse/2016/en) codes. In addition, the Cause of Death Register holds information on all dates and causes of death [42]. Similar registries exist in Norway, Denmark and Finland and have been used in collaborative diabetes research [11, 43].

Everyone who lives in Sweden is automatically reported in many administrative systems, which guarantees a high degree of coverage. However, the data are not primarily intended for research purposes and may be incomplete or incorrect. Nonetheless, the near-complete coverage and very large numbers of people eligible for inclusion in scientific studies present golden opportunities for large-scale studies with long follow-up times and very low dropout rates and superior generalisability.

In RRCTs, linkage to databases from one or more registries is possible with relative simplicity, as all NDR and official databases and registries use the same 12-digit unique individual identifiers. This, in combination with refined statistical methodology, have enabled national and Nordic and European collaborations to be set up to address the associations between diabetes, participant characteristics, risk factors, treatments and procedures, and clinical outcomes [44–49].

## Using a registry-based randomised clinical trial to provide evidence for first-line medication in type 2 diabetes: the SMARTEST trial

Metformin is currently the recommended first-line medication for most patients with type 2 diabetes according to EASD, ADA and most national treatment guidelines [50, 51].

However, the underlying evidence is surprisingly weak and is mainly based on results from a subset of the UK Prospective Diabetes Study (UKPDS) population. In this study, 1704 participants with newly diagnosed type 2 diabetes and with overweight or obesity were randomly assigned to metformin (*n*=342), sulfonylureas (*n*=542), insulin (*n*=409) or diet treatment (*n*=411). Metformin displayed some significant or borderline significant benefits regarding diabetes-related clinical outcomes [52].

Other studies and meta-analyses provide meagre support for metformin as a treatment of choice in early type 2 diabetes, and a Cochrane meta-analysis published in 2020 [53] came to the following striking conclusion: ‘There is no clear evidence whether metformin monotherapy compared with no intervention, behaviour changing interventions or other glucose-lowering drugs influences patient-important outcomes’. Nonetheless, there are also obvious benefits of metformin. It has more than 60 years of widespread clinical use. It is effective for short- and long-term glucose lowering and, when used appropriately, it is safe and well tolerated. It can be combined with all other glucose-lowering agents and, importantly, the daily treatment cost is low.

Taken together, there is a need for head-to-head outcome trials comparing metformin with other agents as first-line medication across subgroups of type 2 diabetes. But which other candidates should be included? In fact, several drug classes may be relevant: dipeptidyl peptidase 4 (DPP-4) inhibitors, sulfonylureas, GLP-1 receptor agonists or SGLT2 inhibitors [54]. Several GLP-1 receptor agonists have shown beneficial effects in preventing major adverse cardiovascular events in type 2 diabetes patients with established CVD or very high cardiovascular risk. In such patients, SGLT2 inhibitors prevent heart failure and renal impairment events. Of note, in these trials the experimental drug or placebo was added on top of standard-of-care glucose-lowering medication and, to a large extent, blood pressure- and lipid-lowering agents [55].

Importantly, there is presently no evidence that currently justifies the broad introduction of SGLT2 inhibitors or GLP-1 receptor agonists in early stage type 2 diabetes, although some guidelines, notably from the European Society of Cardiology, have attempted to markedly widen their recommended usage [56].

### A route towards evidence-based first-line pharmacological treatment

**Fig. 4:**
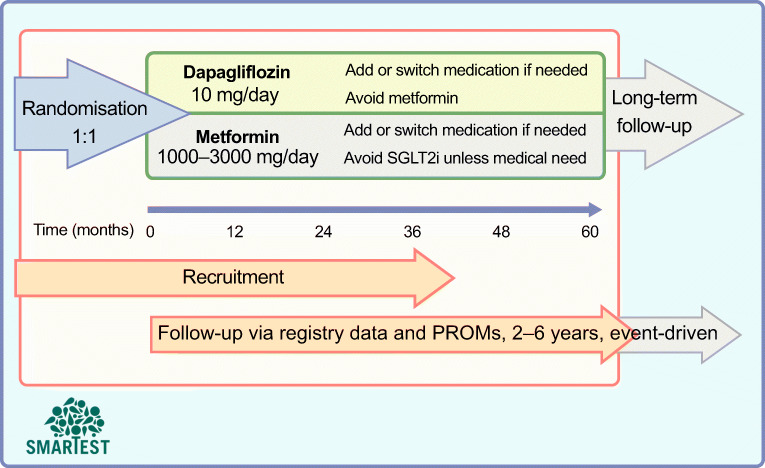
Schematic overview of the SMARTEST trial. Type 2 diabetes patients with less than 4 years since diagnosis are randomly assigned 1:1 to metformin or dapagliflozin treatment. They are followed until 844 events of the primary composite endpoint have occurred, and the time in the study for each participant is estimated to be 2–6 years. Other treatments are according to routine care and glucose-lowering agents can be amended as needed, while avoiding the introduction of either study drug class. PROMs, patient-reported outcome measures; SGLT2i, sodium–glucose cotransporter 2 inhibitors. This figure is available as part of a downloadable slideset

There is a need to fill the knowledge gaps regarding the efficacy of first-line medication in type 2 diabetes, in particular with respect to the prevention of chronic diabetes complications and premature death. There is some support in observational studies for favourable cardiovascular outcomes of SGLT2 inhibitor use compared with metformin [57, 58]; however, there are no ongoing long-term outcome studies sponsored by the life science industry that will provide head-to-head comparisons between metformin and other glucose-lowering agents. Together with several scientific and clinical experts, we therefore designed a national study programme, the SMARTEST trial, to compare treatment with dapagliflozin and metformin in early stage type 2 diabetes patients. This investigator-sponsored academic study started in late 2019, with Uppsala University Hospital as a pilot study site. It is ongoing across Sweden and includes about 30 study sites, mainly in primary care centres but also at a few hospital-based clinical research centres. This is an RRCT in which national healthcare registries are used for the assessment of all clinical outcomes. National and local registries are also used to identify suitable primary care centres and to enhance the recruitment of study participants. Details on the rationale and study design have been published [34] and a brief summary is presented below and in Figs 2, 3 and 4.

### Study population, recruitment, treatments and endpoints

The main eligibility criteria are as follows:

- adult type 2 diabetes patients without CVD or renal impairment;
- less than 4 years since diagnosis of type 2 diabetes;
- taking one oral or no diabetes medication.

Participants are recruited through advertisements or internal NDR or other databases or electronic health records at the individual healthcare centres. The inclusion visit can be performed physically at the nearest study site (often the same as the regular primary care centre) or via a video meeting with a study physician at the coordinating study centre, Uppsala University Hospital. Once informed consent is obtained, eligible patients are randomly allocated to the study treatment (dapagliflozin or metformin) at the inclusion visit or within a maximum of 3 weeks thereafter.

We have developed an electronic informed consent system (https://www.eic.its.uu.se; accessed 1 July 2022) [34] to make trials more cost-effective by also allowing video-based inclusions and further enabling remote monitoring of consent forms. Using digital identifiers that are widely available in Sweden for study participants and trial physicians, the system safeguards many key GCP aspects of the informed consent procedure. Many patients with type 2 diabetes live in rural areas, and video-based inclusion enables people in remote areas to participate. Informed consent can thus be obtained digitally, and screening, including blood sampling and routine clinical examination, is subsequently conducted by local healthcare staff.

Participants are randomly assigned in a 1:1 manner to metformin (1000–3000 mg per day) or dapagliflozin (10 mg once daily) (Fig. 4). Thus, study medication is open label and known to participants, local study site staff and healthcare providers but it is blinded to researchers. Any other ongoing glucose-lowering treatment is discontinued. Routine follow-up is performed in primary care according to clinical guidelines, and clinical and laboratory data are reported to the NDR at least once yearly but usually more frequently.

The composite primary outcome reflects ‘event-free survival’ and is defined as time to the first of any major cardiovascular event or microvascular events (occurrence or progression to the next defined stage) or death. Other endpoints include individual components of the primary outcome (i.e. death, heart failure, myocardial infarction or stroke, retinopathy, nephropathy, lower limb neuropathy or angiopathy); the start of insulin therapy; and cardiovascular risk markers (i.e. HbA_1c_, lipids, urinary albumin-to-creatinine ratio, blood pressure and BMI). Moreover, data on safety, patient-reported outcome measures and the health economy are obtained. All outcomes are collected through the national healthcare registries described above for a preliminary follow-up time of 2–5 years for each participant. For monitoring of safety and total event rates there are quarterly outputs of key events from registries. These data are blinded, although an independent data safety monitoring committee can access unblinded data as required.

In total, 844 events are required in order to achieve 90% power to detect a 20% reduction of risk with one agent vs the other. An intention-to-treat approach is applied for evaluation of treatment differences with respect to the primary composite endpoint. The initial estimate of the number of participants required was 4300, but because of a higher microvascular event rate than expected this number can probably be markedly reduced. This will improve the feasibility of the trial and is helpful for recruitment, which has been much slower than planned because of the COVID-19 pandemic and its accompanying restrictions on society and healthcare. Study medications are distributed by local pharmacies following electronic prescription and are free of cost to participants and healthcare centres. Treatment adherence is monitored both by annual telephone follow-up and using registry data on prescribed drug use. Currently, about 1100 participants are enrolled in the study (May 2022) and over 95% have started randomised treatment.

In addition to the main study, there are also add-on studies involving specific analyses. Thus, some study sites and participants are involved in detailed registry-based and clinical assessments addressing retinopathy, nephropathy, diabetic foot problems, cardiovascular events, autonomic nerve activity, home blood pressure and treatment satisfaction. In a few study sites, participants are invited to undergo repeated sampling and biobanking for analyses of blood biomarkers and, in a subset of participants, analysis of the faecal microbiome. This may help to identify responders and non-responders and contribute to future precision medicine efforts in type 2 diabetes.

Barriers related to infrastructure and resources in primary care are addressed in the SMARTEST trial by the real-world design [34]. However, to facilitate inclusion further, the SMARTEST trial provides a network connecting primary care with academia, with regular communication and the provision of information through digital and physical seminars. We have also developed a model in which general practitioners are encouraged to refer people fulfilling the inclusion criteria to the nearest clinical trial unit for screening, inclusion and randomisation [34]. These units have the infrastructure required for conducting clinical trials according to GCP guidelines, ensuring high quality of the trial.

### Local healthcare databases and digital tools for participant recruitment

In order to facilitate identification of eligible participants and increase recruitment further in the SMARTEST trial, novel digital tools have been used and developed. A software programme (Medrave Software, Sweden) is used to identify patients fulfilling the inclusion criteria from medical records in several primary care centres. Primary care units are encouraged to run the programme to identify eligible patients and to send information by secured email to local recruiting clinical trial units.

As mentioned above, video-based remote visits are a useful option for promoting the inclusion of participants. This requires an electronic informed consent form with validated secure identification and signature functions. Such tools can be generic and facilitate multiple studies or they can be trial-specific. Regardless, the costs of and other barriers to setting them up need to be taken into account early on.

In the SMARTEST trial, monitoring adherence and carrying out follow-up through registers are some of the benefits of the RRCT design, as outlined in Table 1. For example, a head-to-head traditional RCT of 4–5 years’ duration in type 2 diabetes cannot easily be blinded because of the need to add on therapy over time, and frequent travel to sites may hinder participant compliance and increase dropout rates. In this real-world design, follow-up of participants is conducted in primary healthcare and physicians are carefully informed about the study procedures and the treatments allocated to participants. They are further instructed to avoid initiation of metformin or SGLT2 inhibitors (i.e. crossover from one study treatment to the other) unless there is a medical need. Participants are interviewed annually by telephone and adherence to the study medication is reviewed.

## Implementation and impact

There is a large unmet need for RCTs in diabetes care, in particular for the evaluation of treatment strategies and specific pharmacological therapies, but also for the assessment of novel devices and electronic disease monitoring systems. Traditional RCTs are often performed in well-defined but narrow patient samples, and therefore ‘real-world’ trials in broader populations are warranted. The costs of traditional RCTs have risen and RRCTs are therefore appealing both because of their cost-effectiveness and because of the opportunity provided to enrol large representative patient groups. According to our experiences and those from other RRCTs, the direct study cost per participant in RRCTs can be reduced by up to 90% compared with traditional RCTs.

National healthcare registries are gradually evolving in many countries and are easily accessible sources for research on outcome data. Thus, RRCTs in diabetes can be launched in both primary and hospital-based care and will provide further evidence on optimal treatment strategies. Emerging results are likely to influence the development or revision of clinical guidelines and practice. Moreover, RRCTs may potentially provide evidence to support the approval of new medical products or new labelling of approved medicines. However, this remains to be explored and agreed with regulatory agencies, as, to our knowledge, there are currently few precedents [59, 60]. Beyond the evaluation of pharmacological treatments, it is also appealing to consider the RRCT concept for broader utility assessments of other intervention types in diabetes as well as other disease areas. These may include diet, behaviour, health service or policy strategies.

We predict that the RRCT concept will become a very useful tool for testing clinically important hypotheses with reliable and robust endpoints in type 2 diabetes. This concept will also prove to be extremely cost-effective and will enable an increasing number of large and representative clinical trials in diabetes and other disease areas to be carried out.

## Supplementary Information

Slideset of figures (PPTX 367 kb)

## Abbreviations

GCP: Good clinical practice
GLP-1: Glucagon-like peptide-1
NDR: National Diabetes Register
RRCT: Registry-based randomised clinical trial
SGLT2: Sodium–glucose cotransporter 2
SMARTEST: SGLT2 Inhibitor or Metformin as Standard Treatment of Early Stage Type 2 Diabetes
SWEDEHEART: Swedish Web-system for Enhancement and Development of Evidence-based care in Heart disease Evaluated According to Recommended Therapies
