# Supplementary material for: Registry-based randomised clinical trials: a remedy for evidence-based diabetes care?
Source: Diabetologia. 2022 Jul 29;65(10):1575–86. doi: 10.1007/s00125-022-05762-x (PMC9334551; doi:10.1007/s00125-022-05762-x)
Supplement: Supplementary file 1 — (PPTX 367 kb) [file 125_2022_5762_MOESM1_ESM.pptx]

## Slide 1
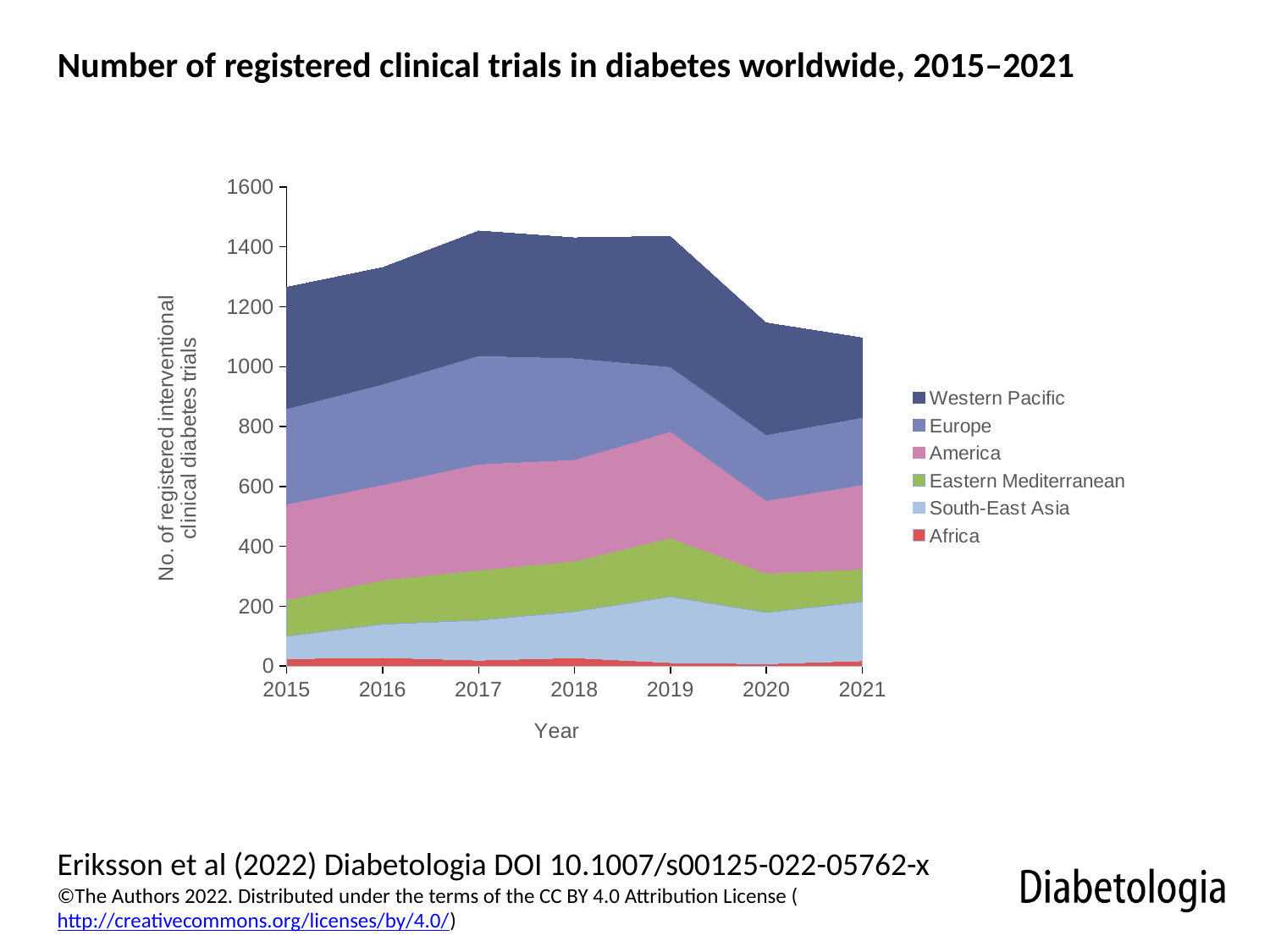

Number of registered clinical trials in diabetes worldwide, 2015–2021
### Chart
| Category | Africa | South-East Asia | Eastern Mediterranean | America | Europe | Western Pacific |
|---|---|---|---|---|---|---|
| 2015 | 25.0 | 75.0 | 122.0 | 319.0 | 319.0 | 405.0 |
| 2016 | 29.0 | 111.0 | 148.0 | 318.0 | 336.0 | 389.0 |
| 2017 | 20.0 | 133.0 | 168.0 | 354.0 | 362.0 | 416.0 |
| 2018 | 28.0 | 154.0 | 169.0 | 339.0 | 339.0 | 401.0 |
| 2019 | 12.0 | 220.0 | 197.0 | 355.0 | 216.0 | 435.0 |
| 2020 | 8.0 | 171.0 | 131.0 | 243.0 | 220.0 | 373.0 |
| 2021 | 18.0 | 197.0 | 109.0 | 282.0 | 225.0 | 265.0 |Eriksson et al (2022) Diabetologia DOI 10.1007/s00125-022-05762-x
©The Authors 2022. Distributed under the terms of the CC BY 4.0 Attribution License (http://creativecommons.org/licenses/by/4.0/)

## Slide 2
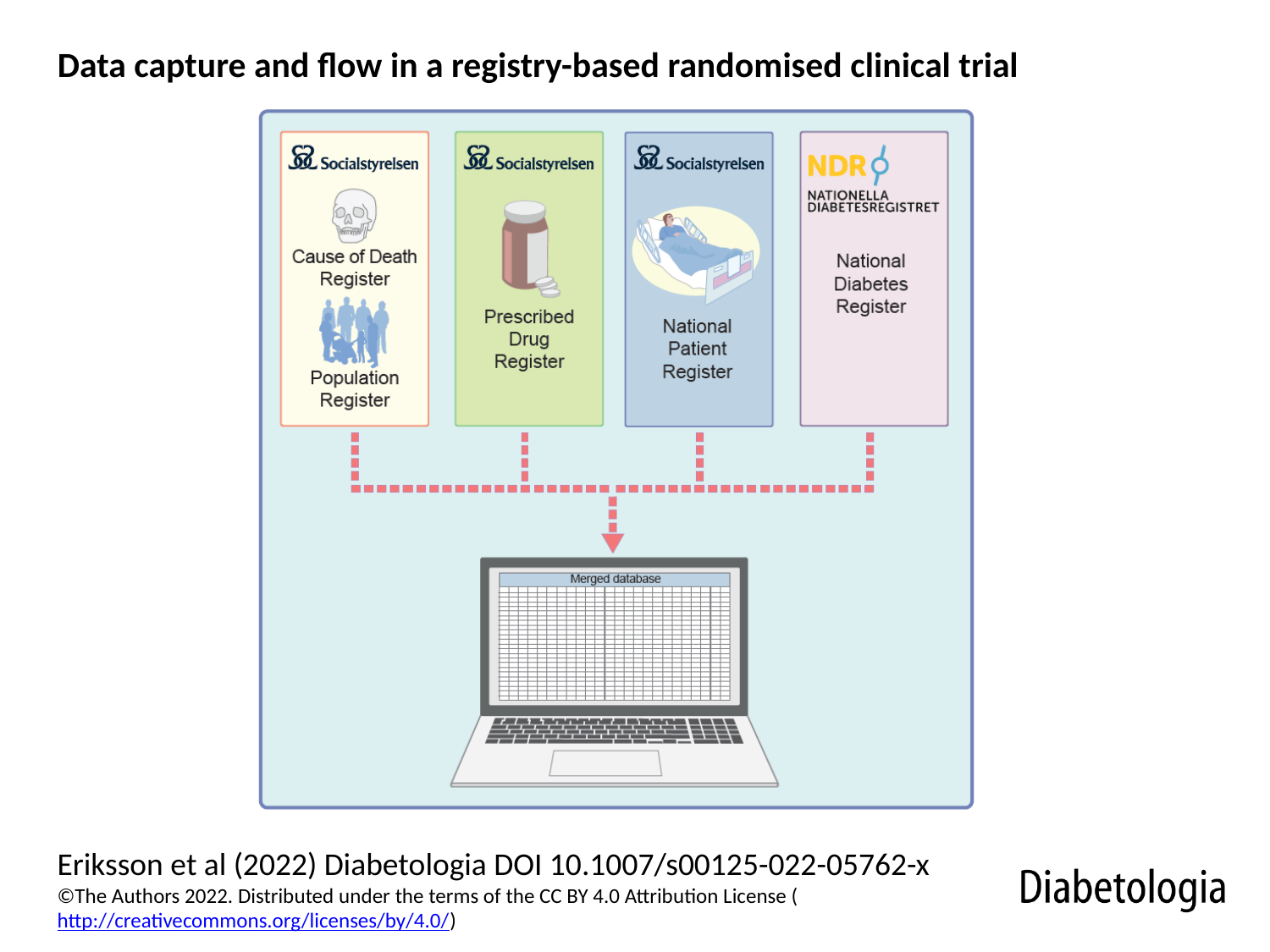

Data capture and flow in a registry-based randomised clinical trial
Eriksson et al (2022) Diabetologia DOI 10.1007/s00125-022-05762-x
©The Authors 2022. Distributed under the terms of the CC BY 4.0 Attribution License (http://creativecommons.org/licenses/by/4.0/)

## Slide 3
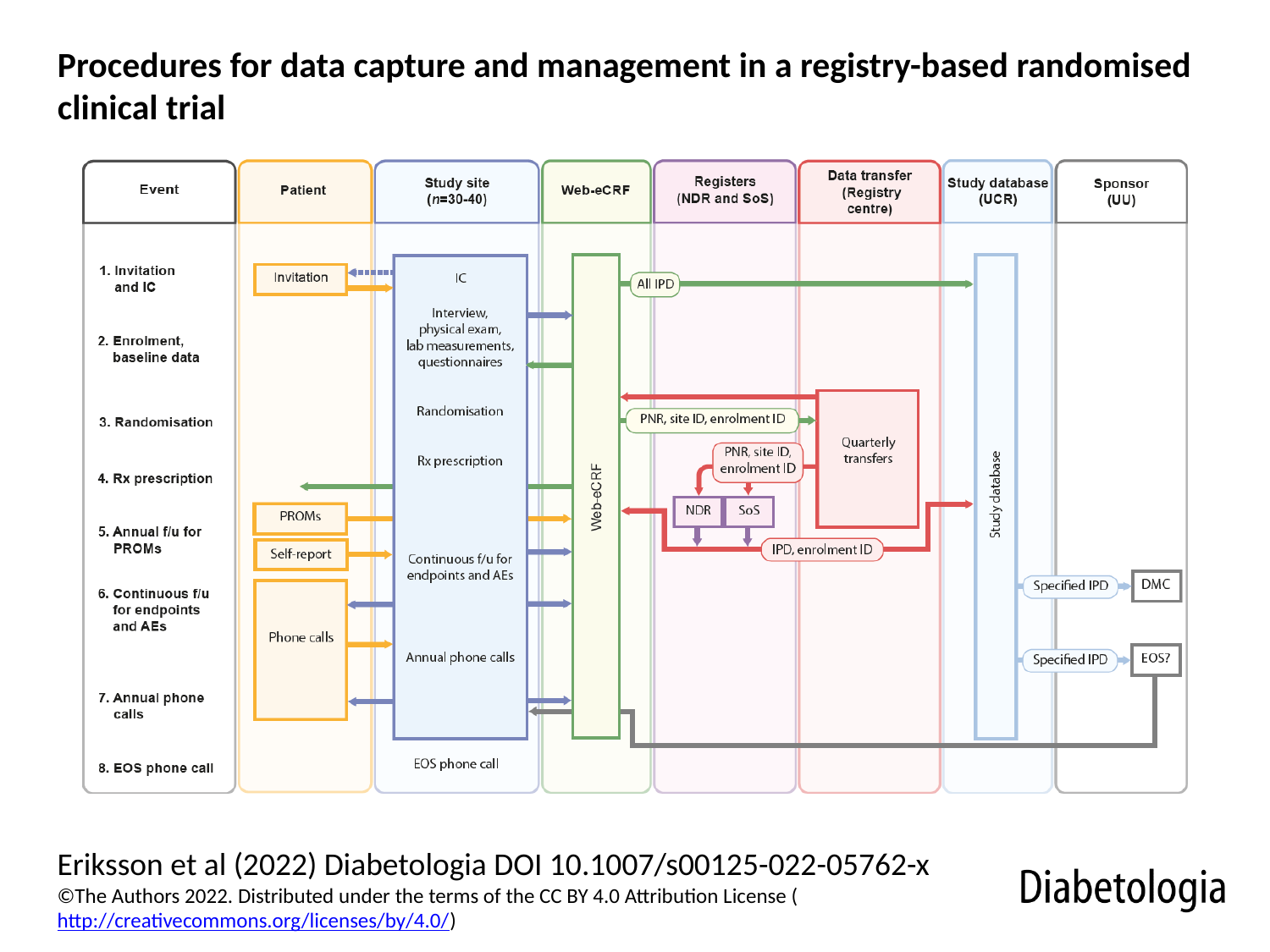

Procedures for data capture and management in a registry-based randomised clinical trial
Eriksson et al (2022) Diabetologia DOI 10.1007/s00125-022-05762-x
©The Authors 2022. Distributed under the terms of the CC BY 4.0 Attribution License (http://creativecommons.org/licenses/by/4.0/)

## Slide 4
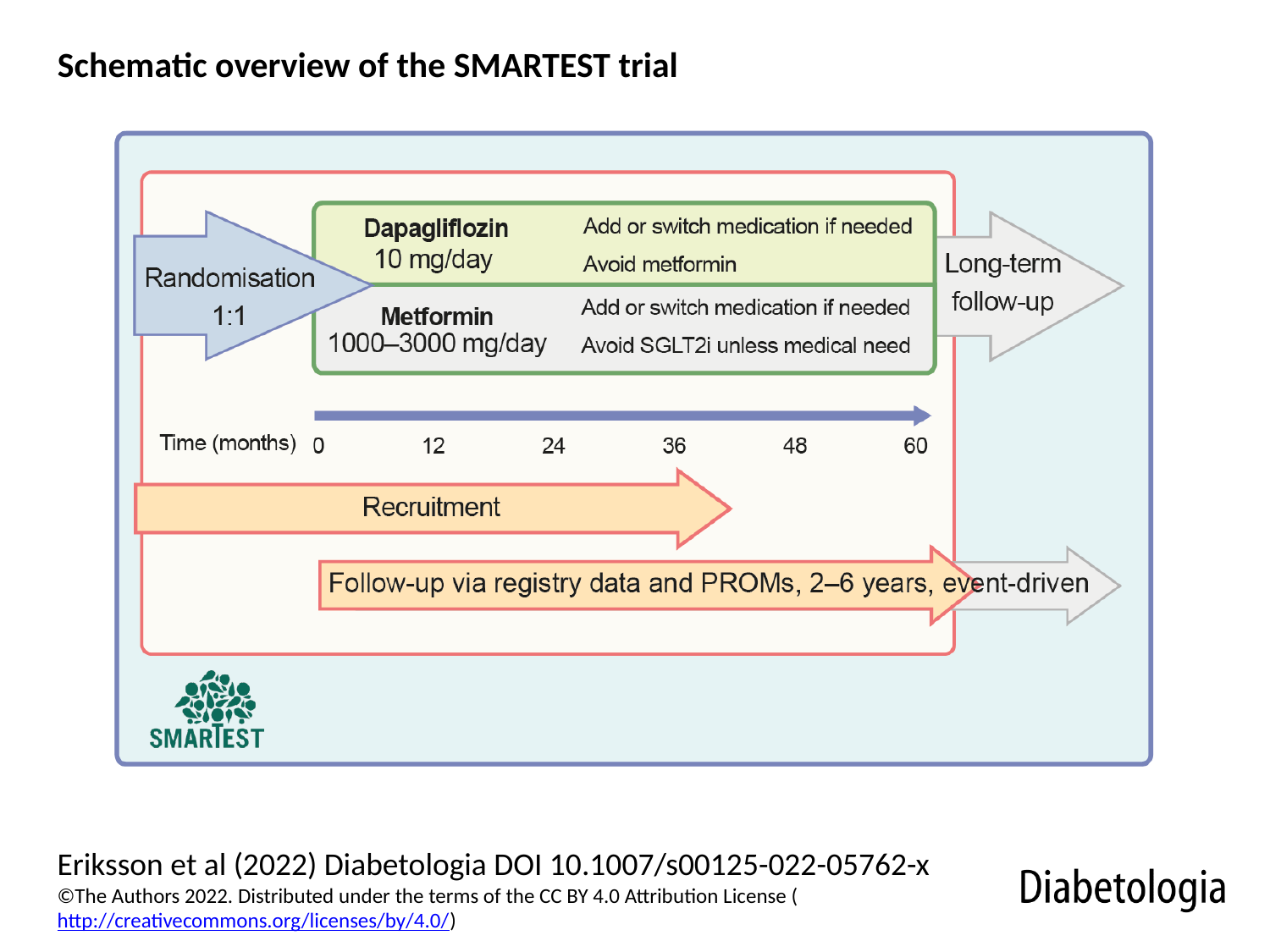

Schematic overview of the SMARTEST trial
Eriksson et al (2022) Diabetologia DOI 10.1007/s00125-022-05762-x
©The Authors 2022. Distributed under the terms of the CC BY 4.0 Attribution License (http://creativecommons.org/licenses/by/4.0/)
